# Computerized Cognitive Training (CCT) versus Yoga Impact on 12 Month Post Intervention Cognitive Outcome in Individuals with Mild Cognitive Impairment

**DOI:** 10.3390/brainsci11080988

**Published:** 2021-07-27

**Authors:** Vaishali S. Phatak, Glenn E. Smith, Dona Locke, Anne Shandera-Ochsner, Pamela M. Dean, Colleen Ball, Gianna Gutierrez, Melanie J. Chandler

**Affiliations:** 1 Department of Neurological Sciences, University of Nebraska Medical Center, Omaha, NE 68131, USA; 2 Department of Clinical and Health Psychology, University of Florida, Gainesville, FL 32610, USA; glennsmith@phhp.ufl.edu (G.E.S.); gianna.gutierrez@ufl.edu (G.G.); 3 Department of Psychiatry and Psychology, Mayo Clinic Arizona, Scottsdale, AZ 85259, USA; Locke.Dona@mayo.edu; 4 Department of Psychiatry and Psychology, Mayo Clinic Health System, La Crosse, WI 54601, USA; Shandera-Ochsner.Anne@mayo.edu; 5 Department of Psychiatry and Behavioral Sciences, University of Washington, Seattle, WA 98108, USA; Pamela.Dean@va.gov; 6 Division of Clinical Trials and Biostatistics, Mayo Clinic Florida, Jacksonville, FL 32224, USA; Thomas.Colleen@mayo.edu; 7 Department of Psychiatry and Psychology, Mayo Clinic Florida, Jacksonville, FL 32224, USA; Chandler.Melanie@mayo.edu

**Keywords:** behavioral interventions, computerized cognitive training (CCT), physical exercise, yoga, mild cognitive impairment (MCI), cognitive enrichment, clinical trial

## Abstract

There is currently limited and mixed evidence for the cognitive benefits of Computerized Cognitive Training (CCT) and yoga in persons with Mild Cognitive Impairment (pwMCI). The objective of this study was to investigate the benefit of computerized cognitive training (CCT) vs. physical (yoga) intervention on cognitive abilities. Participants in this study were part of the larger Mayo Clinic’s Healthy Action to Benefit Independence and Thinking (HABIT) program comparative effectiveness trial. The HABIT program is designed for pwMCI and their care partner and consists of five behavioral interventions: CCT, Memory Support System-Calendar (MSS-Calendar), wellness education, support groups, and yoga. The subtractive study design randomly withheld one of the interventions for a total of five study arms. Longitudinal mixed-effects regression models were used to investigate the hypothesis that CCT and yoga has a greater positive impact on psychomotor and basic attention abilities at 12 months post-intervention as compared to the other HABIT interventions. Findings showed CCT had a positive impact compared to yoga on the Cogstate psychomotor/attention composite at 12 months post-intervention (ES = 0.54; unadjusted *p* value = 0.007, adjusted *p* value = 0.021). The impact of yoga or combining CCT with yoga did not show statistically significant improvement. Continued CCT practice at home showed further benefit on psychomotor/attention at 12 months post-intervention. There was no significant benefit of CCT or yoga on Cogstate learning/working memory composite.

## 1. Introduction

The global population of adults over the age of 65 is expected to grow from 8.5% in 2015 to 16.7% by 2050 [1]. With the increase in proportion of older adults, there is concern for increased cases of Alzheimer’s disease and other related dementias. In 2015, it was estimated that 46 million individuals were living with Alzheimer’s disease globally, and the number of individuals with Alzheimer’s disease is expected to reach as high as 131.5 million by the year 2050 [2]. Given these demographic trends, there is interest in investigating the benefits of salutary cognitive and physical activities in older adults with and without cognitive impairment.

Observational studies have found that cognitively enriching leisure activities, such as reading, playing board games, and playing musical instruments, reduce risk of cognitive impairment in older adults [3]. More detailed research has found that a higher amount of leisure activity (1 h a day) was beneficial for reducing risk of dementia [4]. Meta-analysis of “single-component” or single cognitive domain training and “multicomponent” or multiple cognitive domain training found either training types to be beneficial in healthy older adults as well as persons with Mild Cognitive Impairment (pwMCI) [5]. Ideally, cognitive training can be transferred from narrow trained skills to broader activities. There is some encouraging evidence that working memory training with older adults has shown to transfer to fluid intelligence tasks, processing speed tasks, and episodic memory tasks [6,7,8]. The benefit of cognitive training in adulthood presents exciting avenues of investigation for neuroplasticity.

Computerized cognitive training (CCT) on cognitive functioning in older adults is gaining interest, as CCT interventions have the potential to be disseminated with greater ease. CCT interventions are administered via tablets or personal computers that may be stand-alone software, or web- or app-based. For example, multi-tasking training via video games can improve multi-tasking performance in healthy older adults (60–85 years old) compared to age-matched control groups assigned to single-task training or no-contact [9]. The Advanced Cognitive Training for Independent and Vital Elderly (ACTIVE) trial had domain-specific training in memory, reasoning, and processing speed delivered via CCT, and showed maintenance effects with better processing speed and reasoning performance compared to memory in healthy older adults at a 10 year follow-up [10]. CCT is flexible enough to be conducted in the lab or at home with the participant’s own equipment. CCT with in-lab training and at home for healthy adults (65+) and healthy adults aged 50–64 showed small to middle effect size improvements in visual attention and processing speed [11,12].

CCT is generally tolerated in pwMCI. For example, an intensive at-home CCT program of 100 min per day, 5 days a week for 6 weeks, found that withdrawal rates for pwMCI receiving CCT vs. pwMCI in the control group to be comparable. In the pwMCI receiving CCT, 5 of 22 pwMCI withdrew from the study compared to 6 of 27 pwMCI in the control group [13].

Despite the tolerability of CCT in pwMCI, there are few studies investigating the impact of CCT on cognitive functioning in MCI. One review that limited inclusion to randomized control trials with at least 12 weeks of CCT found only eight qualified studies. Due to the limited pool, the overall conclusion was indeterminate regarding the slowing of dementia progression or impact on cognitive performances [14]. Given this limitation, there is a need for further methodical investigation on the benefit computerized cognitive training.

Similar to CCT, there is a growing body of literature suggesting the benefit of physical exercise on cognitive functioning. A meta-analysis of 13 RCT studies found there was a benefit in global cognitive functioning as measured by MMSE on individuals with Alzheimer’s disease [15]. Studies evaluating the benefit of physical exercise for pwMCI have been mixed, with evidence for improvement on global cognitive functioning and inconsistent benefits observed across other cognitive domains [16,17]. A systematic review and meta-analysis of 25 RCT that were classified as aerobic, resistance training or Tai-Chi found that resistance training showed benefits compared to stretching/toning for the reasoning measure. Whereas, Tai Chi showed benefits compared to “no exercise” controls on measures of attention and processing speed [18]. A systematic review with meta-analysis of 11 studies with yoga intervention in individuals aged over 55 and over OR Individuals ≥ 55 years old found benefits in multiple cognitive domains including memory, executive function, attention, and processing speed [19].

Meta-analyses of combined cognitive and physical exercise interventions have also shown benefits in global cognitive outcome in individuals with MCI and dementia [20,21].

The goal of the present analysis was to investigate the impact of CCT and yoga on cognitive functioning for pwMCI following participation in a modified version of Mayo Clinic’s Healthy Action to Benefit Independence and Thinking (HABIT) program.

HABIT is a five-component program that includes CCT, yoga, MSS-Calendar, wellness education, and support groups. Of the 272 dyads who enrolled in the study, 237 dyads completed procedures at a 6 month follow-up, 228 completed procedures at a 12 month follow-up, and 218 participants at a 18 month follow-up [22]. We hypothesized that each of the HABIT interventions would provide different types of benefits for pwMCI and their care partner. For example, we hypothesized that pwMCI receiving support group intervention would benefit quality of life and self-efficacy but not directly benefit objective cognitive outcome. Similarly, we hypothesized that receiving MSS-Calendar training would allow participants to utilize their calendar in daily life and benefit self-efficacy, but the calendar training was not expected to transfer to objective cognitive outcome. Somewhat unexpectedly, the results showed the greatest effect size for quality of life was between the pwMCI group that did not receive wellness education compared to the pwMCI group that did not receive CCT [23]. Similarly, the findings showed that suppressing wellness education or yoga had a negative impact on the care partner’s anxiety level [24].

We hypothesized that CCT and yoga would both independently and when combined provide beneficial impact to psychomotor and basic attention at 12 months post-training in pwMCI. We further hypothesized that the benefit of CCT and yoga as independent and combined interventions would be more evident on psychomotor/attention outcomes, as both cognitive training and physical exercise have a greater potential to improve psychomotor speed and basic attention compared to learning/working memory outcomes. We did not anticipate any transfer of benefit to objective cognitive outcome from the other HABIT interventions (i.e., MSS-Calendar, wellness education, and support groups).

## 2. Materials and Methods

Participants in this study were part of the larger trial of comparative effectiveness of behavioral interventions in pwMCI from Mayo Clinic’s HABIT (Healthy Action to Benefit Independence and Thinking) program. The study was approved by the Institutional Review Board of Mayo Clinic (ID# 14-000885) and the University of Washington, Seattle, WA, USA, (ID# 49235). This study followed Consolidated Standards of Reporting Trials reporting guidelines.

Dyads (pwMCI and their care partners) were recruited at four sites (Mayo Clinic, Rochester, Minnesota; Mayo Clinic, Scottsdale, Arizona; Mayo Clinic, Jacksonville, Florida; University of Washington, Seattle, WA, USA) from September 2014 to December 2016 for participation in the intervention trial. Inclusion criteria for pwMCI were a clinical diagnosis of amnestic or multi-domain MCI and a global rating of ≤0.5 on the Clinical Dementia Rating (CDR) [25]. CDR is a structured interview used to assess the stage of dementia that ranges from 0 to 3, with 0 representing no cognitive decline and 3 representing severe dementia. Further inclusion criteria included not taking medication or taking stable nootropic medications for at least three months, English fluency, and a care partner with a Mini-Mental Status Exam [26] score of >24 with at least twice weekly contact with pwMCI. Exclusion criteria for participation were marked auditory or visual impairment that would preclude participation in interventions or participation in another intervention clinical trial. Full details of the trial can be found in the protocol description [27]. A total of 272 pwMCI consented to participate in the study. Individuals included for the purposes of the present study had a valid available baseline and 12 months cognitive outcome data.

HABIT is delivered in a group-based format. For that reason and to prevent treatment diffusion, all participants in any one session received the same four behavioral interventions. During each session, one of the five interventions was randomly suppressed; participants were unaware of the suppressed intervention until day 1 of the program. Each of the study sites ran all five study arms at least once. Each behavioral intervention consisted of 60 min of training for a total of 4 h of intervention per day for 10 consecutive weekdays. Participants received a “booster” session at 6 months after the intervention program and at 12 months after the intervention program. The “booster” sessions at both of those time points lasted one day and repeated the four behavioral interventions that were included in the participant’s initial training.

(1) The CCT intervention consisted of training on the Posit Science product BrainHQ™ (www.brainhq.com, accessed on 1 September 2014) tasks using tablets during a two-week training period. Participants were provided with a one-year subscription to BrainHQ to access with their personal devices at home after the initial two-week training session. Brain HQ consists of various tasks across a variety of cognitive domains, including attention, processing speed, memory, decision making, visuospatial navigation, and recognition. During the two-week training the participants explored and trained on multiple Brain HQ tasks for 60 min each day. After the two-week training, participants were advised to continue 150 min of CCT per week for 12 months post training. Adherence to cognitive training was tracked through access provided by Posit Science for study investigators. (2) Yoga groups were led by certified yoga instructors. Participants engaged in a 60 min session of chair and standing yoga poses as well as breathing and meditative exercises. Participants were encouraged to perform 150 min of physical activity per week at home. Participants were provided with a DVD of the yoga poses and exercises for continued practice at home. (3) MSS-Calendar sessions consisted of 60 min of training for pwMCI to incorporate the use of a calendar with three sections: appointments, to-do list, and notes. Structured training questions graduated participants along three phases (acquisition, application, and adaptation) with the end goal of having the MSS-Calendar be a reliable compensatory device for the participant’s everyday use. (4) Wellness education consisted of a 60 min group lecture with a different topic each day of the two-week training. Topics were relevant for pwMCI, such as sleep hygiene, roles and relationships, nutrition, assistive technologies, etc. (5) Support groups for pwMCI and partners were conducted separately. The group for pwMCI had 60 min reminiscence-focused group sessions with the goal of processing a diagnosis of MCI and its impact. The care partner group had a 60 min session with caregiving themes, such as role changes, communication, disclosure to family/friends, caregiver health, etc.

Cognitive outcome of the behavioral interventions was measured using the Cogstate brief battery (CBB). CBB has been validated to assess the trajectory of cognitive change in healthy adults and pwMCI [28,29]. Composite scores for psychomotor/attention and learning/working memory derived from four subtests have been useful in detecting cognitive change in MCI and mild–moderate Alzheimer’s dementia [30].

The four CBB tasks (Detection (DET), Identification (IDN), One Card Learning (OCL) and One Back Task (ONB)) were administered at baseline and at 12 months post-intervention. Study staff oriented the participant to the Cogstate battery and provided supervision during practice trials. Study staff administering the Cogstate battery were blind to the study arm of the participant.

Detection (DET) is a reaction time task where the participant touches “Yes” when they see the playing card presented on the screen has been turned over. If the participant touch “Yes” when the card is not flipped, it is considered an error. The task yields the primary score of reaction time in milliseconds and a secondary score of accuracy.

Identification (IDN) is a task of visual attention and reaction time where the participant is shown playing cards and asked to respond to the question “is the card red?” Participants have a dichotomous choice of a “Yes” or “No” response. The task yields the primary score of reaction time in milliseconds for accurate responses and a secondary score of accuracy.

One Card Learning (OCL) is a visual attention and learning task. Participants are shown various playing cards and asked to respond to the question “have you seen this card before in this task?” Participants have a dichotomous choice of a “Yes” or “No” response. The task yields the primary score of accuracy and a secondary score for reaction time in milliseconds.

One Back Task (ONB) is a task of working memory. Participants are shown various playing cards and asked to respond to the question “is the previous card the same?” Participants have a dichotomous choice of a “Yes” or “No” response. This task yields both reaction time and accuracy.

The four tasks yielded reaction time and accuracy scores provided by Cogstate. The reaction times in milliseconds were transformed on the logarithm base 10 scale for DET, IDN, and ONB. Accuracy, measured by the proportion of correct responses, was transformed by taking the arcsine square root of the proportion correct for the OCL and ONB tasks.

The psychomotor/attention composite score was derived through the following steps: (1) IDN and DET speed scores were standardized against within-sample baseline values and transformed by multiplying by −1 so that higher scores indicate better performance. (2) The psychomotor/attention composite score is then calculated by taking the average of the DET and IDN standardized scores [28]. The learning/working memory composite score was based on OCL and ONB accuracy scores. The learning/working memory composite score was calculated by taking the average of standardized score of OCL and ONB scores [28]. The primary outcome of the study was performance on a psychomotor/attention composite score at 12 months follow up after intervention.

Longitudinal mixed-effects regression models were used to evaluate the impact of CCT and yoga on cognitive scores at 12 months compared to baseline for the psychomotor/attention composite cognitive score and the learning /working memory composite cognitive score. Mean change in the cognitive score was modelled with fixed effects for three groups, including the no yoga arm, the no CCT arm, and the combined no MSS-Calendar and no support groups, which each included both CCT and yoga interventions, age, and sex. As it was not expected that MSS-Calendar, wellness education or support groups would directly impact cognition and to reduce multiple comparisons, we used linear contrasts from each model to evaluate three comparisons of interest regarding their impact on cognitive scores at 12 months: CCT versus yoga, CCT without yoga versus CCT with yoga, and yoga without CCT versus yoga with CCT.

For evaluating the impact of CCT versus yoga on cognitive scores at 12 months, we focused on estimation of the difference (d) between the average of the means in the no CCT arm (μ1) and the mean in the no yoga arm (μ2) by expressing it as a linear contrast: d = −1μ1 + 1μ2 + 0μ3 + 0μ4 + 0μ5, where μ3, μ4, and μ5 are the means for the arms that included both CCT and yoga. For the comparison of CCT without yoga to CCT with yoga, we estimated the difference between μ2 and the average of the means in the three arms that included both CCT and yoga, where d = 0μ1 + 1μ2 − 0.33μ3 − 0.33μ4 − 0.33μ5. In a similar fashion, we compared yoga without CCT to yoga with CCT by estimating the difference between μ1 and the average of μ3, μ4, μ5, where d = 1μ1 + 0μ2 − 0.33μ3 − 0.33μ4 − 0.33μ5. We constructed 95% confidence intervals (CI) using the profile likelihood method and corresponding likelihood ratio tests. We adjusted for multiple testing with the Holm method for adjustment. Analyses were performed using R statistical software, version 3.6.2 (R Foundation for Statistical Computing).

## 3. Results

The present analysis was conducted with individuals who had available Cogstate data at baseline and at 12 months follow-up from the larger comparative effectiveness trial that consisted of 272 pwMCI. Table 1 provides demographic characteristics of the baseline sample with Cogstate data.

At baseline, there were no meaningful differences in baseline scores for the psychomotor/attention composite score, the learning/working memory composite score, or individual subtest scores between intervention groups (see Table 2).

**Figure 1:**
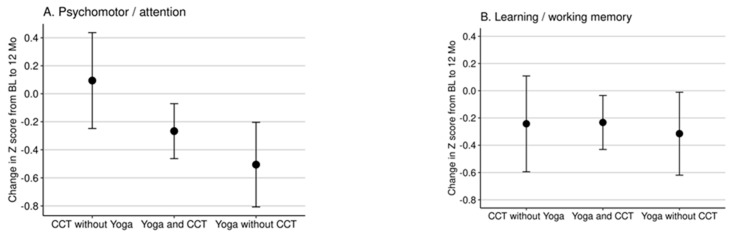
(**A**) Impact of CCT and yoga on psychomotor/visual attention outcome; (**B**) impact of CCT and yoga on learning/working memory outcome.

CCT had a positive impact compared to yoga on the psychomotor/attention composite at 12 months post-intervention (Figure 1A; ES = 0.54; unadjusted *p* value 0.007, adjusted *p* value = 0.021). CCT without Yoga showed a greater benefit compared to CCT with Yoga but was not statistically significant after adjustment for multiple comparison (ES = 0.32; adjusted *p* value = 0.11). Yoga without CCT compared to Yoga with CCT was also not statistically significant after adjustment of multiple comparison (Table 3).

There was no statistically significant difference for CCT vs. Yoga on learning/working memory (Figure 1B; Table 3).

At-home CCT adherence after the supervised intervention showed a median of 180 min of participation over the course of 12 months. Total raw block time on the at-home CCT post-supervised intervention was used as a continuous measure in a log base 10 format. Subsequent analysis found that increased total raw block time on at-home CCT post-intervention was associated with improvement in the psychomotor/attention composite (z score = 0.215; 95% CI = 0.015 to 0.412, *p* = 0.035) at 12 months.

## 4. Discussion

The primary goal of this study was to investigate the benefit of CCT and yoga on objective cognitive outcomes in pwMCI. Our study found that a two-week, supervised intervention of CCT had a positive impact (ES = 0.54) on psychomotor/attention at 12 months follow-up. The benefit of CCT was not seen in learning/working memory. Unexpectedly, yoga did not show benefit on either psychomotor/attention or learning/working memory, nor did combining yoga with CCT further improve cognitive outcomes.

The apparent benefits of CCT on psychomotor/attention in pwMCI were consistent with previous literature of CCT in pwMCI. It is somewhat surprising that neither the yoga nor the combined CCT and yoga intervention showed robust benefits on cognitive outcomes. In our previous findings, yoga showed benefits for memory-related activities of daily living [23]. The literature on physical exercise interventions has shown positive impacts on cognition, including processing speed [31,32]. It is possible that the present mixed findings could be due to limited transfer from training to broader tasks. Although the CCT intervention (BrainHQ) was a multi-component intervention rather than treating a single cognitive domain, many of the tasks within BrainHQ had a processing speed component and as such may have led to improved task-specific skills rather than a generalized cognitive improvement. Another possible explanation could be the type of physical exercise (i.e., yoga vs. aerobic) used in the present intervention. Aerobic exercise may potentially contribute to greater psychomotor improvement or overall cognitive benefit compared to stretching/toning exercises. Finally, another consideration and limitation of the present study is that it is more easily possible for individuals who had physical activity withheld to supplement their intervention by participating in outside physical activities.

Participants who received CCT intervention were prescribed to engage in 150 min per week of at-home CCT for the 12 months following the intervention. However, as we have reported previously, the majority of the participants in the study did not continue to engage in the prescribed target at-home CCT [22]. Nevertheless, it is encouraging that there were greater benefits in psychomotor/attention at the 12 months follow-up for pwMCI who participated in greater amounts of at-home CCT. In addition, an important area for future research would be the investigation of modifiable factors or interventions to help improve at home adherence to CCT.

The research design employed for the present study had limitations, including the subtractive design, which did not allow for a control group that did not receive intervention. Another limitation is that because of informed consent, participants were aware that they were missing an intervention and they may have actively sought the withheld intervention outside the formal training. A drawback of the CCT was that there was no standardized curriculum during the sessions. Participants were allowed to explore any of the cognitive tasks within BrainHQ during the 60 min session. Individuals may have had biased approaches when choosing tasks.

## 5. Conclusions

A two-week CCT intervention with pwMCI showed benefit at 12 months outcome with greater benefit in individuals who continued to participate in CCT after the initial two-week training. While the cognitive benefit was narrowly confined to psychomotor/attention and did not transfer to learning/working memory, it is encouraging that there was benefit in cognitive training in older adults. The lack of benefit from physical training (yoga) or combined physical and cognitive training was surprising. With greater physical longevity, it is important for us to further understand the benefits of modifiable lifestyle factors, such as engagement in cognitively and physically challenging experiences.

## Figures and Tables

**Table 1:** Participant demographics at baseline by study arm.

Composite Score	No Yoga (N = 31)	No CCT (N = 43)	No Wellness Education (N = 40)	No Support Groups (N = 35)	No MSS-Calendar (N = 34)
Age, mean (SD)	75.4 (7.5)	75.7 (8.6)	77.1 (7.0)	74.5 (8.4)	74.2 (7.9)
Male %	64.5	60.5	60.0	68.6	52.9

**Table 2:** Patient Cogstate composite scores at baseline and 12 months follow-up.

Composite Score	No Yoga	No CCT	No Wellness Education	No Support Groups	No MSS−Calendar	Baseline *p* Value
**Psychomotor/attention**						
Baseline, mean (SD)	−0.073 (0.905)	0.036 (0.868)	−0.196 (0.969)	0.072 (0.910)	0.174 (0.797)	0.32
12 months, mean (SD)	−0.008 (1.010)	−0.446 (1.033)	−0.640 (1.243)	−0.263 (0.924)	−0.066 (1.058)	
**Learning/working memory**						
Baseline, mean (SD)	0.133 (1.027)	0.068 (0.823)	−0.200 (0.807)	0.157 (0.749)	−0.130 (0.848)	0.32
12 months, mean (SD)	−0.145 (1.004)	−0.197 (1.199)	−0.174 (0.867)	−0.258 (0.942)	−0.002 (0.909)	

Abbreviations: CCT, computerized cognitive training; MSS-Calendar, memory support system. Likelihood ratio test *p* values evaluating for potential differences in baseline scores between study arms result from linear mixed effects regression models with random effects for study site and fixed effects for study arm, patient age, and patient sex.

**Table 3:** Impact of CCT and yoga on patient cognitive composite scores at 12 months post-intervention outcome.

Composite	Contrasts	Difference at 12 mo (95% CI)	Original *p* Value	Adjusted *p* Value
**Primary outcome**				
Psychomotor/Attention	CCT vs. Yoga	0.54 (0.15 to 0.92)	0.007	0.021
	CCT without Yoga vs. CCT with Yoga	0.32 (−0.00 to 0.65)	0.053	0.11
	Yoga without CCT vs. Yoga with CCT	−0.21 (−0.51 to 0.09)	0.17	0.17
**Secondary outcome**				
Learning/ Working memory	CCT vs. Yoga	0.06 (−0.31 to 0.44)	0.75	1.00
	CCT without Yoga vs. CCT with Yoga	−0.01 (−0.33 to 0.31)	0.96	1.00
	Yoga without CCT vs. Yoga with CCT	−0.07 (−0.36 to 0.22)	0.64	1.00

Abbreviations: CI = confidence interval; CCT = Computerized Cognitive Training. Both original and multiple-test adjusted *p* values are shown.

## Data Availability

Data are available from the corresponding author upon reasonable request.
